# [^68^Ga]NOTA-Galactosyl Human Serum Albumin: a Tracer for Liver Function Imaging with Improved Stability

**DOI:** 10.1007/s11307-017-1046-1

**Published:** 2017-02-13

**Authors:** Roland Haubner, Andreas M. Schmid, Andreas Maurer, Christine Rangger, Llanos Geraldo Roig, Bernd J. Pichler, Irene J. Virgolini

**Affiliations:** 1 Department of Nuclear Medicine, Medical University of Innsbruck, Anichstr. 35, 6020 Innsbruck, Austria; 2 Werner Siemens Imaging Center, Department of Preclinical Imaging and Radiopharmacy, Eberhard Karls University Tübingen, Röntgenweg 13, 72076 Tübingen, Germany

**Keywords:** Liver function imaging, Galactosyl human serum albumin, Nota, Gallium-68, Positron emission tomography

## Abstract

**Purpose:**

Non-invasive techniques allowing quantitative determination of the functional liver mass are of great interest for patient management in a variety of clinical settings. Recently, we presented [^68^Ga]DTPA-GSA to target the hepatic asialoglycoprotein receptor for this purpose. Here, we introduce [^68^Ga]NOTA-GSA to improve metabolic stability of the radiopharmaceutical and compare the imaging properties with [^68^Ga]DTPA-GSA.

**Procedures:**

Labeling of the compounds was carried out at room temperature using 1.9 M sodium acetate as buffer. For quality control, thin-layer, high-performance liquid, and size exclusion chromatographies were used. Metabolic stability was studied in rat and human serums. For *in vivo* evaluation, Fischer rats were scanned by positron emission tomography and magnetic resonance imaging and subsequently sacrificed for biodistribution studies. Time activity curves (TACs) for heart and liver were generated and corresponding parameters (T_50_, T_90_, LHL15, HH15) were calculated.

**Results:**

[^68^Ga]NOTA-GSA can be produced in high radiochemical yield and purity (>95 %) within 15 min. Stability studies revealed almost no metabolite formation over the 2-h observation period. Analysis of the TACs showed comparable results for most of the investigated parameters. The only significant difference was found in the T_90_ value, where [^68^Ga]NOTA-GSA showed slower uptake in comparison with ^68^Ga-DTPA-GSA (123 ± 10 vs. 89 ± 3 s, *p* < 0.01).

**Conclusions:**

[^68^Ga]NOTA-GSA showed a significant increase of the metabolic stability and in most organs lower background activity. However, comparison of LHL15 and HH15 indicates that the increased stability did not further improve the diagnostic value. Thus, [^68^Ga]NOTA-GSA and [^68^Ga]DTPA-GSA can be used equivalent for imaging hepatic function with positron emission tomography.

## Introduction

During the last decade, an increasing interest in the use of gallium-68 with positron emission tomography (PET) has been observed. This is mainly based not only on the improving availability of corresponding Ge-68/Ga-68 generators [1] but also due to the very convincing results obtained with Ga-68-labeled radiopharmaceuticals like [^68^Ga]DOTATOC (see, e.g., [2]) and [^68^Ga]HBED-PSMA (see, e.g., [3]). Thus, gallium-68 became an interesting alternative to fluorine-18 in labeling compounds for imaging with PET.

On the other hand, non-invasive methods allowing quantitative determination of the functional liver mass are of great interest for patient management in a diversity of clinical settings comprising liver surgery and liver transplantation [4–6] as well as diagnosis [7, 8] and therapy monitoring [9] of cancer. Additionally, it has been revealed that the evaluation of remnant liver function can help to discriminate different stages of alcoholic liver cirrhosis [10] and could be used to differentiate areas of steatosis, fibrosis, and cholestasis [11]. Moreover, control of liver status before and during peptide receptor radionuclide therapy (PRRT) [12] could lead to an optimized patient management. Furthermore, patients who are potentially suitable for selective internal radiation therapy (SIRT) [13] may also benefit from such a diagnostic method, allowing stratifying patients according to their peri-interventional risk.

[^99m^Tc]diethylenetriamine-pentaacetic acid galactosyl human serum albumin ([^99m^Tc]GSA) [14] binds to the asialoglycoprotein receptor (ASGP-R), a hepatic cell surface receptor specific for galactose terminated glycoproteins [4]. This receptor is exclusively expressed on the sinusoidal surface of mammalian hepatocytes. It has been proven that [^99m^Tc]GSA and dynamic single-photon emission computed tomography (SPECT) allows estimation of regional hepatic function based on the determination of the ASGP-R density [15, 16].

To combine the superior performance of PET compared with SPECT concerning imaging resolution and quantifying properties with the good properties of GSA in ASGP-R targeting, we developed a Ga-68-labeled analog [17]. [^68^Ga]DTPA-GSA showed comparable targeting properties as found for [^99m^Tc]GSA but lack of high metabolic stability. Thus, in this study, we introduce with 2-S-(4-isothiocyanatobenzyl)-1,4,7-triazacyclononane-1,4,7-triacetic acid (p-NCS-Bn-NOTA) an alternative chelating moiety and present stability data as well as the PET imaging properties of [^68^Ga]NOTA-GSA and compare them with [^68^Ga]DTPA-GSA.

## Material and Methods

All reagents were obtained from VWR International GmbH (Vienna, Austria) or Sigma-Aldrich Handels GmbH (Vienna, Austria) and were used without further purification. DTPA-conjugated galactosyl human serum albumin (GSA) was a kind gift from Nihon Medi-Physics (Tokyo, Japan) and was supplied as a freeze-dried technetium kit formulation. The kit was reconstituted with water and directly used for ^68^Ga labeling without isolation of the glycoprotein. The mean number of DTPA groups per human serum albumin (HSA) is 6 and the mean number of galactosyl units is 35 resulting in an estimated MW of 80,730 g/mol (for details, see [17]). NOTA-GSA was supplied from piCHEM (Graz, Austria) and includes six NOTA, conjugated via isothiocyanate chemistry and 19 galactosyl moieties per HSA molecule resulting in an estimated MW of 76,398 g/mol. The Ge-68/Ga-68 generator was purchased from Eckert & Ziegler (Berlin, Germany) with a nominal activity of 1100 MBq and was eluted with 0.1 N HCl (Biochemical grade, FLUKA, Switzerland).

### Labeling of DTPA-GSA and NOTA-GSA with Ga-68

For both compounds, Ga-68 labeling was carried out using a fractionated elution protocol [18]. Synthesis of [^68^Ga]DTPA-GSA followed the protocols published in [17]. Briefly, 100 μg DTPA-GSA (dissolved with water to 1 mg/ml using the freeze-dried kit formulation) and 30 μl 1.9 M sodium acetate solution was incubated for 30 min with 300 μl [^68^Ga]GaCl_3_ in 0.1 M HCl (50–100 MBq) at room temperature. In analogy, for labeling of NOTA-GSA 100 μg precursor (dissolved with water to 4 mg/ml) and 30 μl 1.9 M sodium acetate solution were incubated for 15 min with 300 μl [^68^Ga]GaCl_3_ in 0.1 M HCl (approx. 50 MBq) at room temperature.

For PET imaging studies, the ratio between precursor amount and Ga-68 activity used was kept constant (1 μg per MBq). Reaction was carried out at room temperature in 172 mM sodium acetate buffer for 30 min. This results in specific activities of approx. 83 MBq/nmol for [^68^Ga]DTPA-GSA and approx. 77 MBq/nmol for [^68^Ga]NOTA-GSA in the animal studies.

For standard quality control, thin-layer chromatography (TLC) and high-performance liquid chromatography (HPLC) was used. TLC was carried out using Varian iTLC-SG (Palo Alto, CA, USA) and 0.1 M sodium citrate pH 5. For analysis of the TLC strips, a phosphor imager was used (Cyclone Plus Storage Phosphor System, PerkinElmer, Waltham, MA, USA). For HPLC a Dionex Ultimate 3000 RS HPLC system with pump, column compartment, and variable wavelength UV detector (Thermofischer Scientific, Vienna, Austria) and a Gabi Star radiometric detector (Raytest, Straubenhardt, Germany) were used. A Vydac 218TP5215 C18 polymeric reversed phase column (5 μm, 300 Å, and 150 × 3.0 mm; SRD, Vienna, Austria), flow rates of 1 ml/min, and UV detection at 220 nm were employed with the following acetonitrile (ACN)/H_2_O/0.1 % trifluoro acetic acid (TFA) gradient: 0–2 min 10 % ACN, 2–20 min 10–60 % ACN, 20–21 min 60–100 % ACN, and 21–26 min 100 % ACN.

Additionally, size exclusion chromatography (SEC) was carried out to control for potential colloid formation. Therefore, Sephadex G-25 PD-10 columns (GE Healthcare Europe GmbH, Vienna, Austria) were incubated with 15 ml bovine serum albumin solution (BSA, 1 % in isotonic saline), loaded with 0.2 ml reaction mixture, and washed with 7 ml isotonic saline. Eluate and column were measured in a dose calibrator.

### Stability of [^68^Ga]DTPA-GSA and [^68^Ga]NOTA-GSA

The metabolic stability in human serum was determined after 2, 30, 60, and 120 min incubation of [^68^Ga]NOTA-GSA at 37 °C. Hundred microliter [^68^Ga]NOTA-GSA labeling solution per 1 ml serum were used for incubation. For HPLC analysis, 400 μl samples of the human serum were passed through a 0.20 μm sterile filter (Millex LG, EMD Millipore, Bellerica, MA; activity remaining on the sterile filter was between 1 and 2 %) and 20 μl aliquots were injected to the HPLC (gradient A). Stability assay for [^68^Ga]DTPA-GSA (gradient B) in human serum was carried out following the same protocol and is described in [17]. Assays were carried out in duplicate.

The metabolic stability of [^68^Ga]NOTA-GSA and [^68^Ga]DTPA-GSA in rat serum was determined after 2, 30, and 60 min at 37 °C. Therefore, 20 μl of the corresponding tracer was added to 200 μl of serum. At the corresponding time points, 20 μl aliquots were removed and diluted with 80 μl water, passed through a 0.20 μm sterile filter, and the filter was washed with 100 ml water. Eighty microliter samples were analyzed using HPLC and gradient A. Assays were carried out in duplicate.

ACN/H_2_O/0.1 % TFA gradients were 0–2 min 10 % ACN, 2–20 min 10–60 % ACN, 20–21 min 60–100 % ACN, and 21–26 min 100 % ACN (gradient A) and 0–2 min 0 % ACN, 2–18 min 0–80 % ACN, 18–19 min 80–100 % ACN, and 19–23 min 100 % ACN (gradient B).

### Imaging and Biodistribution Studies

For the *in vivo* comparison of [^68^Ga]NOTA-GSA and [^68^Ga]DTPA-GSA, two groups of healthy male Fischer rats (*n* = 4 per group, 253 ± 11 g) were measured in a sequential PET/MRI setup. Animals were anesthetized in 1.5 % isoflurane evaporated in medical oxygen and placed on the PET Scanner (Inveon dedicated PET, Siemens Healthcare, Knoxville, TN, USA). After injection of the corresponding tracer (4.9 ± 0.1 MBq of [^68^Ga]NOTA-GSA and 5.1 ± 0.1 MBq of [^68^Ga]DTPA-GSA) animals were measured dynamically for 30 min followed by a transmission scan for attenuation correction. PET data were reconstructed using OSEM2D reconstruction algorithm including the attenuation correction. For an anatomical scan (3D RARE, TR/TE = 300/29 ms) the animals were transferred on the animal bed without moving the animal to a 7T MRI (BioSpec, Bruker Biospin GmbH, Ettlingen, Germany).

For biodistribution studies, blood samples were taken and animals were sacrificed immediately after imaging studies were finished. Organs (liver, lung, kidneys, spleen, heart, gut, stomach, and brain) as well as muscle were excised. The samples were weighed and activity in the samples was measured using a γ-counter (Wizard single-detector-counter; PerkinElmer, Waltham, MA, USA). Results are expressed as the percentage injected dose per gram of tissue (%ID/g). Each value represents the mean and SD of four animals. All animal experiments were approved by the German competent authorities (Regierungspräsidium Tübingen; R12/13).

For analysis, the images of PET and MRI were fused and regions of interest were drawn on the basis of the MRI, covering the entire liver and the left ventricle for the cardiac input curve using Inveon Research Workplace (IRW, Siemens Healthcare). Time activity curves (TACs) were analyzed and characterizing parameters were calculated using MatLab (Mathworks, Natick, MA, USA). Briefly, a second-degree polynomial was fitted into the five points around the maximal value of the cardiac input function, defining *t*_0_ for the experiment. Subsequently, a decreasing exponential function *y* = *A* ⋅  exp (−*b* ⋅ *x*) + *c* was fitted into the cardiac TAC, starting at the peak value of the polynomial. Liver uptake was also characterized by an exponential function, but was fitted over the entire dataset. Based on these functions, characteristic parameters for blood clearance (*T*_50_ = time to reach 50 % of the maximum heart uptake; HH15 = blood activity at 15 min divided by the blood activity at 3 min; decaying constant *b* of the fit) and functional liver reserve (T_90_ = time to reach 90 % of the maximum liver uptake; LHL15 = liver activity at 15 min divided by the sum of liver and blood activity at 15 min) were calculated.

## Results

### Synthesis of [^68^Ga]NOTA-GSA

**Fig. 1.**
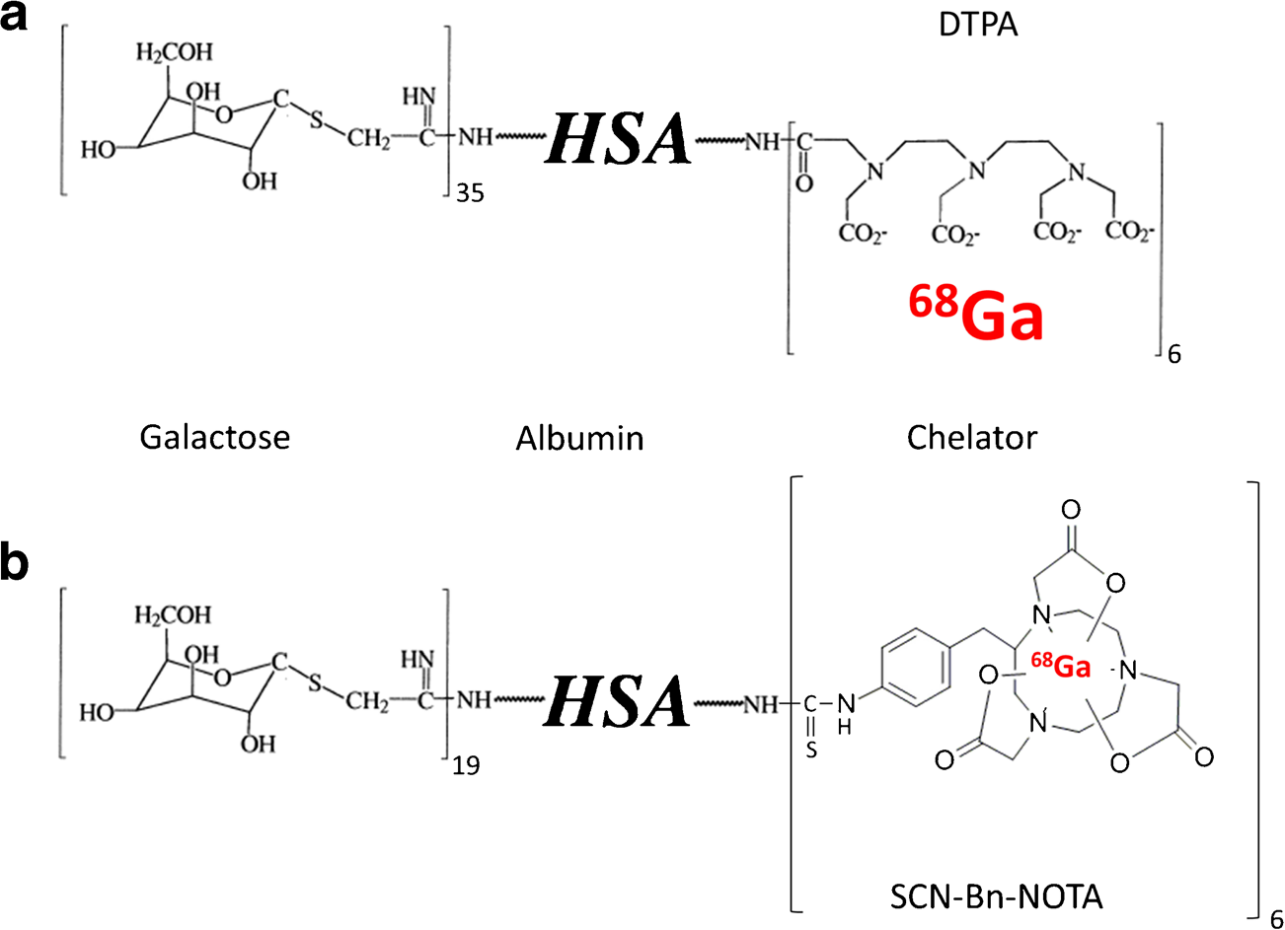
Schematic structure of **a** [^68^Ga]DTPA-GSA and **b** [^68^Ga]NOTA-GSA. Differences are found in the chelator type and the amount of sugar conjugated to the human serum albumin (HSA).

Labeling of NOTA-GSA (for proposed structure, see Fig. 1) with gallium-68 could be carried out in radiochemical yield and purity >95 % determined by HPLC as well as TLC. Additional analysis of potential colloid formation using SEC demonstrated that the formulation contains >97 % [^68^Ga]NOTA-GSA and indicating that if at all only low amounts of colloid had been formed. The specific activities of the studied preparations were in the range of 38–77 MBq per nmol NOTA-GSA. Due to the fact that no separation of the unlabelled compound is possible, the specific activity strongly depends on the amount of precursor used.

### Stability Assays

**Fig. 2.**
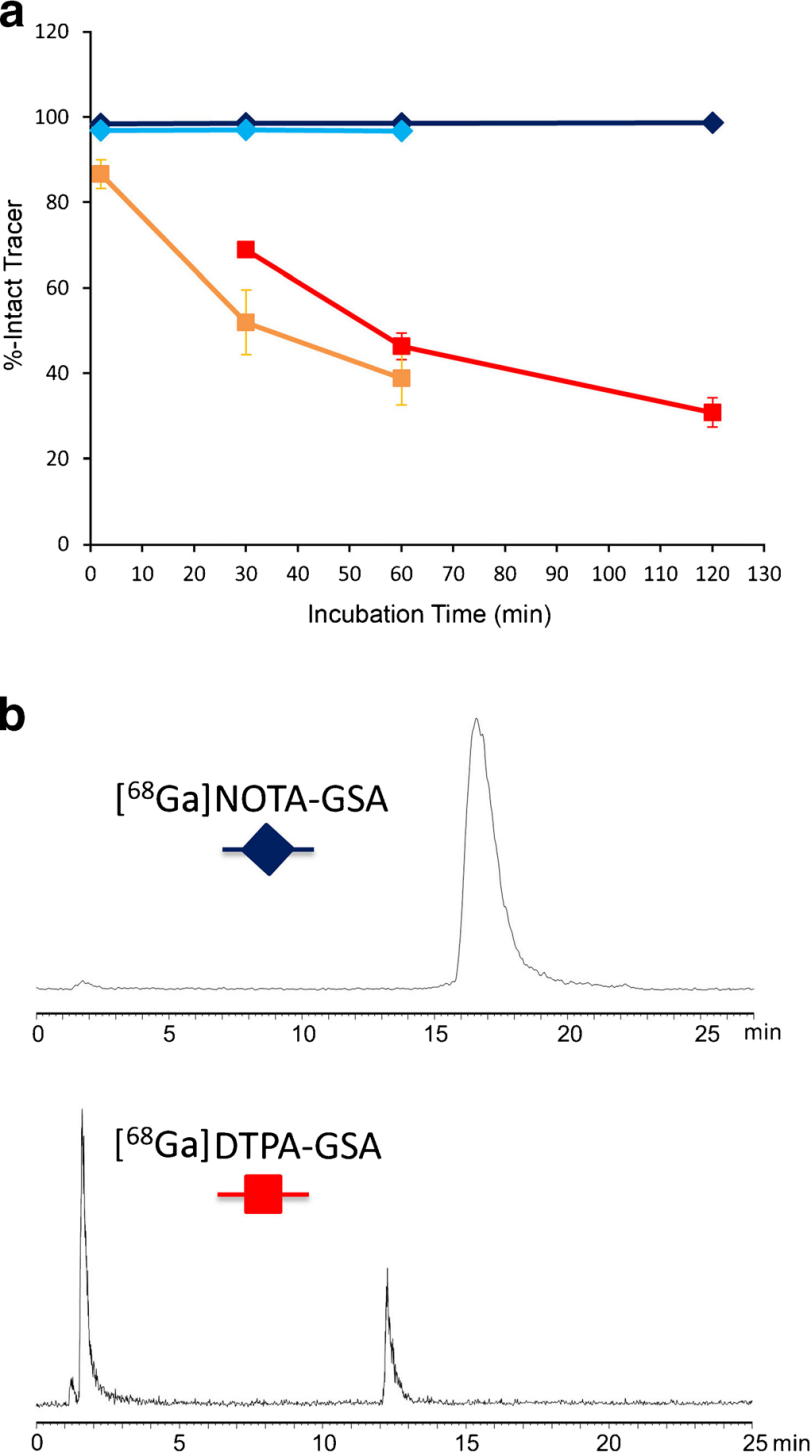
**a** Comparison of the metabolic stability of [^68^Ga]NOTA-GSA (*blue*) and [^68^Ga]DTPA-GSA (*red*) in human serum after 30-, 60-, and 120-min incubation at 37 °C (for [^68^Ga]NOTA-GSA additional samples were taken approx. 2 min after start of incubation) and comparison of the stability in rat serum ([^68^Ga]NOTA-GSA (*light blue*), [^68^Ga]DTPA-GSA (orange)) after 2-, 30-, and 60-min incubation at 37 °C. Studies were carried out in duplicate (for [^68^Ga]NOTA-GSA standard deviation is very low and cannot be visualized due to the size of the used symbols). **b** HPLC diagrams of the different compounds after 120-min incubation in human serum. The retention times of the intact tracers are 16.6 min (gradient A) and 12.3 min (gradient B) for [^68^Ga]NOTA-GSA and [^68^Ga]DTPA-GSA, respectively.

Incubation of [^68^Ga]NOTA-GSA with human serum up to 120 min demonstrated high metabolic stability of the tracer (Fig. 2). Over the whole observation period of 120 min, more than 98 % intact tracer was found if samples were analyzed via HPLC. In contrast, rapid degradation was found for ^68^Ga-DTPA-GSA. Already after 30-min incubation, only approx. 70 % intact tracer was found. After 120 min, the amount of intact tracer was further reduced to 30 %. Due to the low retention time, it can be assumed that the observed “degradation product” is mainly due to release of the radiometal from the chelating system.

The same behavior is found when analyzing the metabolic stability in rat serum. Again, [^68^Ga]NOTA-GSA was stable over the observation period of 60 min (imaging data where recorded not longer than 30 min; thus for the rat study, observation time was reduced) whereas a significant reduction of intact [^68^Ga]DTPA-GSA was observed. After 1-h incubation, approx. 97 % of intact [^68^Ga]NOTA-GSA was found compared with approx. 39 % for [^68^Ga]DTPA-GSA (see Fig. 2).

#### Comment

Due to the physiological role of the asialoglycoprotein receptor, which transfers the glycoproteins into liver cells where they are degraded stability analysis in the liver would not supply evaluable information about the stability of the chelating systems and were thus not included.

### Imaging and Biodistribution Data

**Fig. 3.**
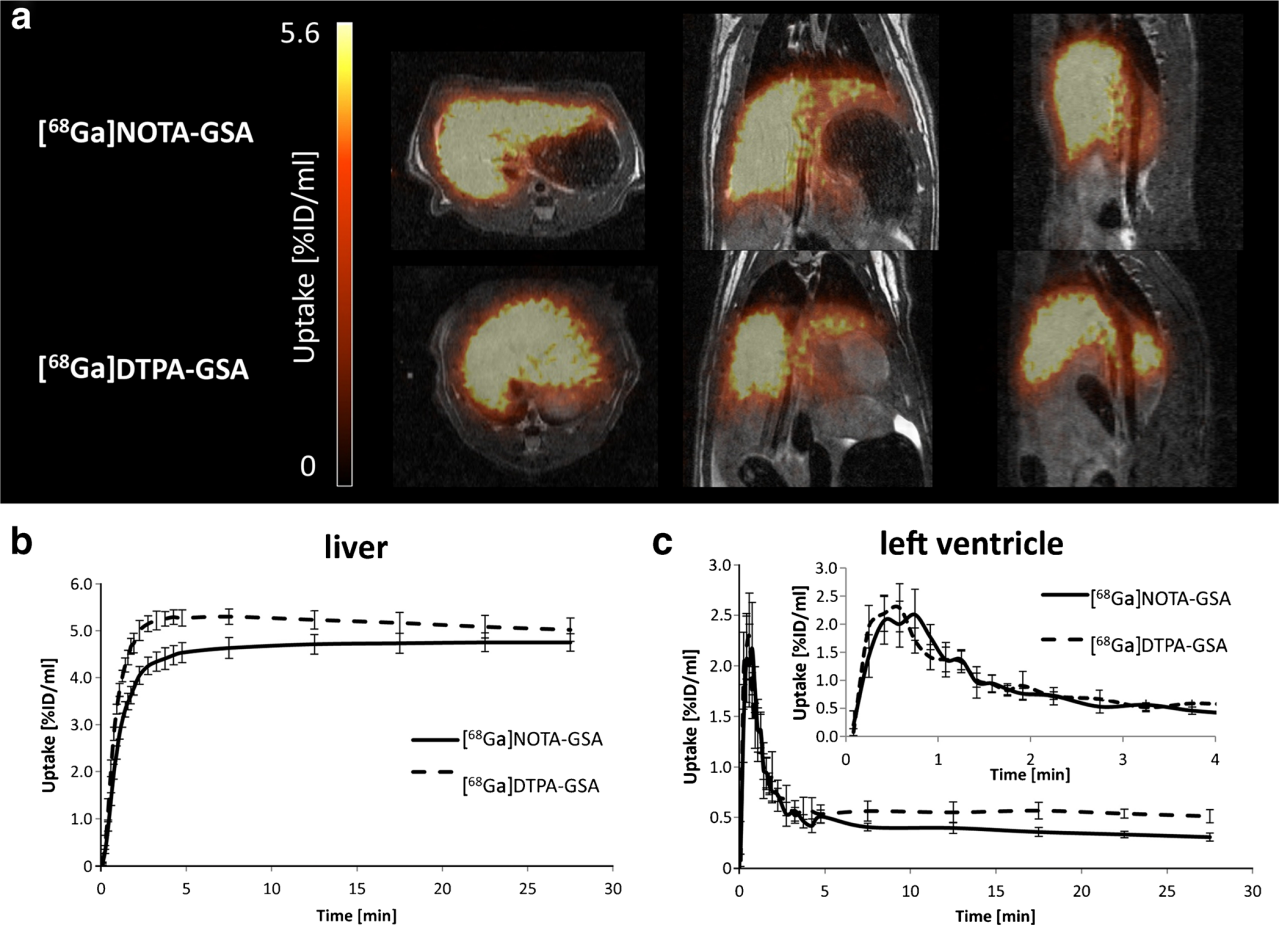
Comparison of [^68^Ga]NOTA-GSA and [^68^Ga]DTPA-GSA accumulation *in vivo*. In representative images **a** both compounds yielded comparable results 30 min post injection. High uptake in the liver is found while in all other organ activity concentration was negligible. At first glance time activity curves (**b**, **c**) were comparable. However, [^68^Ga]DTPA-GSA reached a slightly higher uptake followed by a wash-out in the liver (**b**) and higher background activity in the blood pool (**c**) compared to [^68^Ga]NOTA-GSA.

**Fig. 4.**
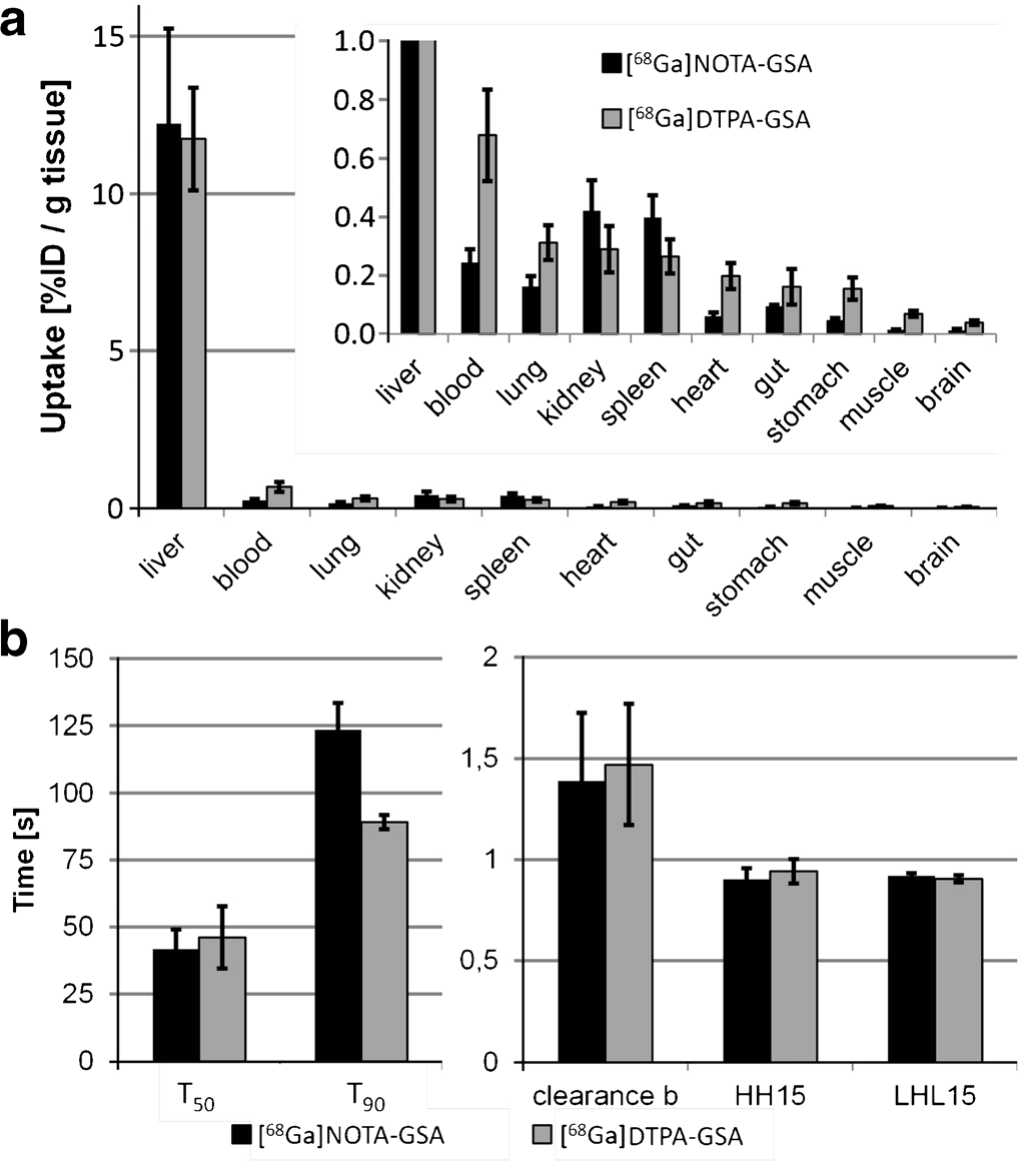
Comparison of [^68^Ga]NOTA-GSA and [^68^Ga]DTPA-GSA ex vivo. *In vivo* imaging results were verified by biodistribution studies, which were performed approximately 35 min after the PET emission scan, leading to a further reduction of liver uptake in the [^68^Ga]DTPA-GSA group. **a** Again, both tracers showed high accumulation in the liver with only minor activity in all other investigated organs. **b** Analysis of time activity curves revealed increased *T*_90_ times for [^68^Ga]NOTA-GSA; however, all other calculated parameters were identical.

Both tracers showed comparable uptake patterns, i.e., a very fast accumulation throughout the entire liver with low background activity in all other organs and a very fast blood clearance (Fig. 3). In direct comparison, [^68^Ga]DTPA-GSA reached a slightly higher liver uptake level, but also showed a slow decrease over time (Fig. 3a). Mean TACs generated at the left ventricle were very similar, showing a very fast decrease to baseline in the first 5 min with [^68^Ga]NOTA-GSA reaching a lower final level (Fig. 3c). The biodistribution confirmed the *in vivo* results, showing only relevant uptake in liver tissue, while all other organs remained below 0.5 % ID/g tissue (Fig. 4a). In-depth analysis of the TACs, based on exponential fits, also showed comparable results for most of the investigated parameters. Overall fit-quality was excellent (*R* ^2^ for liver TAC is 0.99 ± 0.01 and for blood TAC 0.94 ± 0.03 averaged over all animals). Most parameters were comparable (Table 1). The only significant difference was found in the *T*_90_ value, where [^68^Ga]NOTA-GSA showed slower uptake in comparison with [^68^Ga]DTPA-GSA (123 ± 10 vs. 89 ± 3 s, *p* < 0.01).

**Table 1:** Characteristic parameters for *blood clearance* (T_50_ = time to reach 50 % of the maximum heart uptake, HH15 = blood activity at 15 min divided by the blood activity at 3 min, and decaying constant b of the fit) and *functional liver reserve* (T_90_ = time to reach 90 % of the maximum liver uptake and LHL15 = liver activity at 15 min divided by the sum of liver and blood activity at 15 min). Data are given as mean ± standard deviation

Compound	*T*_50_ [s]	*T*_90_ [s]	Clearance b	HH15	LHL15
[^68^Ga]NOTA-GSA	42 ± 7	123 ± 10	1.39 ± 0.33	0.90 ± 0.05	0.92 ± 0.01
[^68^Ga]DTPA-GSA	46 ± 12	89 ± 3	1.47 ± 0.30	0.94 ± 0.06	0.90 ± 0.02

## Discussion

[^99m^Tc]GSA and dynamic SPECT including data analysis using corresponding kinetic models [19] has successfully been demonstrated to be able to determine non-invasively regional hepatic function in a great number of patients especially in Japan [14]. Recently, we introduced the Ga-68-labeled analog [^68^Ga]DTPA-GSA and showed that the technetium kit formulation can directly be labeled with Ga-68 [17]. Moreover, the new tracer showed comparable liver uptake as found for the ^99m^Tc-analog. However, the stability in human serum was inferior to [^99m^Tc]GSA. This might be due to the use of DTPA as gallium chelating system. In this study, we replaced the DTPA moiety by a NOTA derivative and could demonstrate that this modification resulted in a much more stable GSA derivative ready for imaging liver function in clinical settings.

Initially, DTPA-GSA was designed to be labeled with Tc-99 m [20], for which DTPA is a well-established chelator. It is known that DTPA possesses also a high complex stability constant for Ga(III) [21]. Thus, it could be assumed that the corresponding [^68^Ga]DTPA-GSA complexes should be sufficiently stable for non-invasive imaging of the hepatic function. However, in our previous study, we found significant degradation in human serum within 30 min [17]. The low stability of ^68^Ga-DTPA complexes is confirmed by Anderson and Strand [22] who studied the stability of a Ga-67-labeled monoclonal antibody and found also low stability of the complex. To improve the metabolic stability of the tracer, we replaced the DTPA moiety by a NOTA derivative. It is known that the nine-membered ring structure of NOTA forms a cage with an optimal size for complexing Ga(III), resulting in a very high complex stability constant [21].

Synthesis was carried out to produce the labeling precursor with a similar average amount of chelating moieties as found for the DTPA-GSA. Subsequent labeling of NOTA-GSA was straightforward. Within 15 min at room temperature, [^68^Ga]NOTA-GSA can be produced in high radiochemical purity and yield. No subsequent purification is required. However, it has to be mentioned that fixing of the product, independent if NOTA-GSA or DTPA-GSA is used, on a C-18 cartridge is not possible. Thus, it is recommended to use a pre-purification procedure for labeling to guarantee that no germanium breakthrough can reach any patient preparation in a clinical setting.

Corresponding stability assays carried out either in human or in rat serum both clearly demonstrated the desired improvement. [^68^Ga]NOTA-GSA showed almost no release of the radiometal over the observation period of 2 h in human serum. Even the clinically established [^99m^Tc]DTPA-GSA revealed, over the whole observation period, more degradation as found for [^68^Ga]NOTA-GSA [17]. However, the imaging studies comparing the activity distribution and pharmacokinetics of both ^68^Ga-labeled compounds reveal, at first glance, only small differences in tracer uptake in the liver and in the elimination of the tracer from the body. Anyway, a more detailed analysis revealed that for [^68^Ga]NOTA-GSA activity concentration in most organs beside the liver is even lower as the already low values found for [^68^Ga]DTPA-GSA (e.g., activity concentration in blood for [^68^Ga]NOTA-GSA is less than half of the concentration found for [^68^Ga]DTPA-GSA). Additionally, TACs show a decrease in the liver uptake of [^68^Ga]DTPA-GSA at later time points, whereas it remains constant for [^68^Ga]NOTA-GSA. All this might be attributed to the lower stability of [^68^Ga]DTPA-GSA. However, determination of the relevant parameter based on the TACs resulted in comparable values. An exception is the *T*_90_ value. Due to the fact that the initial uptake of [^68^Ga]DTPA-GSA is faster and also slightly higher than found for ^68^Ga-NOTA-GSA, this value is smaller for [^68^Ga]DTPA-GSA. It seems that the higher stability due to the introduced NOTA system reduces the activity concentration in blood and may slow down the tracer kinetics. As the *T*_90_ time may be more sensitive to physiological parameters such as, e.g., blood flow, general cardiac output, or depth of anesthesia, a relevant influence on the determination of the hepatic function is not expected based on this slight difference, especially since the LHL15 values are almost the same. Therefore, if using the entire dataset to calculate functional liver reserve (LHL15) and blood clearance (HH15), both tracers yield identical results. Thus, the difference in metabolic stability does not significantly affect the final outcome. As the parameters LHL15 and HH15 were developed in a clinical setting using SPECT imaging [23], they may not be the best suitable parameters to characterize the fast metabolism of rats or mice. A recent study by Schnabl et al. investigated the functional liver reserve in an animal model of liver disease using *T*_90_ values to characterize [^68^Ga]DTPA-GSA uptake [24]. Here, we see small differences in the absolute values; however, their influence on the sensitivity has to be investigated in studies with corresponding disease models.

## Conclusion

[^68^Ga]NOTA-GSA can be easily produced in high radiochemical purity and yield within 15-min reaction time. The introduction of the NOTA moiety resulted in a significant increase of the metabolic stability and in most organs in lower background activity. However, comparison of LHL15 and HH15 indicates that the increased stability did not further improve the diagnostic value. Thus, [^68^Ga]NOTA-GSA and [^68^Ga]DTPA-GSA can be used as an equivalent for imaging hepatic function with PET.

## References

[1] Decristoforo C (2012). Gallium-68—a new opportunity for PET available from a long shelf-life generator—automation and applications. Curr Radiopharm.

[2] Froeling V, Elgeti F, Maurer MH (2012). Impact of Ga-68 DOTATOC PET/CT on the diagnosis and treatment of patients with multiple endocrine neoplasia. Ann Nucl Med.

[3] Haberkorn U, Eder M, Kopka K (2016). New strategies in prostate cancer: prostate-specific membrane antigen (PSMA) ligands for diagnosis and therapy. Clin Cancer Res.

[4] de Graaf W, Bennink RJ, Vetelainen R (2010). Nuclear imaging techniques for the assessment of hepatic function in liver surgery and transplantation. J Nucl Med.

[5] Hoekstra LT, de Graaf W, Nibourg GA (2013). Physiological and biochemical basis of clinical liver function tests: a review. Ann Surg.

[6] Kaibori M, Ha-Kawa SK, Maehara M (2011). Usefulness of Tc-99m-GSA scintigraphy for liver surgery. Ann Nucl Med.

[7] Virgolini I, Muller C, Klepetko W (1990). Decreased hepatic function in patients with hepatoma or liver metastasis monitored by a hepatocyte specific galactosylated radioligand. Br J Cancer.

[8] Kurtaran A, Li SR, Raderer M (1995). Technetium-99m-galactosyl-neoglycoalbumin combined with iodine-123-Tyr-(A14)-insulin visualizes human hepatocellular carcinomas. J Nucl Med.

[9] Virgolini I, Kornek G, Hobart J (1993). Scintigraphic evaluation of functional hepatic mass in patients with advanced breast cancer. Br J Cancer.

[10] Virgolini I, Muller C, Angelberger P (1991). Functional liver imaging with 99Tcm-galactosyl-neoglycoalbumin (NGA) in alcoholic liver cirrhosis and liver fibrosis. Nucl Med Commun.

[11] Bennink RJ, Tulchinsky M, de Graaf W (2012). Liver function testing with nuclear medicine techniques is coming of age. Semin Nucl Med.

[12] Mansi L, Virgolini I (2011). Diagnosis and therapy are walking together on radiopeptides' avenue. Eur J Nucl Med Mol Imaging.

[13] D'Arienzo M, Chiaramida P, Chiacchiararelli L (2012). 90Y PET-based dosimetry after selective internal radiotherapy treatments. Nucl Med Commun.

[14] Kokudo N, Vera DR, Makuuchi M (2003). Clinical application of TcGSA. Nucl Med Biol.

[15] Kudo M, Todo A, Ikekubo K (1993). Quantitative assessment of hepatocellular function through *in vivo* radioreceptor imaging with technetium 99m galactosyl human serum albumin. Hepatology.

[16] Vera DR, Stadalnik RC, Metz CE (1996). Diagnostic performance of a receptor-binding radiopharmacokinetic model. J Nucl Med.

[17] Haubner R, Vera DR, Farshchi-Heydari S (2013). Development of Ga-68-labelled DTPA galactosyl human serum albumin for liver function imaging. Eur J Nucl Med Mol Imaging.

[18] Breeman WA, de Jong M, de Blois E (2005). Radiolabelling DOTA-peptides with 68Ga. Eur J Nucl Med Mol Imaging.

[19] Miki K, Kubota K, Inoue Y (2001). Receptor measurements via Tc-GSA kinetic modeling are proportional to functional hepatocellular mass. J Nucl Med.

[20] Stadalnik RC, Vera DR (2001). The evolution of (99m)Tc-NGA as a clinically useful receptor-binding radiopharmaceutical. Nucl Med Biol.

[21] Reichert D, Lewis J, Anderson C (1999). Metal complexes as diagnostic tools. Coordination Chem Rev.

[22] Anderson WT, Strand M (1985). Stability, targeting, and biodistribution of scandium-46- and gallium-67-labeled monoclonal antibody in erythroleukemic mice. Cancer Res.

[23] Ha-Kawa SK, Tanaka Y, Hasebe S (1997). Compartmental analysis of asialoglycoprotein receptor scintigraphy for quantitative measurement of liver function: a multicentre study. Eur J Nucl Med.

[24] Schnabl B, Farshchi-Heydari S, Loomba R (2016). Staging of fibrosis in experimental non-alcoholic steatohepatitis by quantitative molecular imaging in rat models. Nucl Med Biol.

